# Performance of metagenomic next-generation sequencing for bloodstream infections in perioperative critically ill patients- a *post-hoc* analysis of a prospective, multi-center cohort study

**DOI:** 10.3389/fcimb.2026.1814969

**Published:** 2026-06-15

**Authors:** Qiao Qin, Ya-Chan Ning, Sai-Nan Zhu, Jia-Hui Ma, Wei Chen, Wei Tian, Chun-Mei Wang, Ying-Feng Wu, Shuang-Ling Li

**Affiliations:** 1 Department of Critical Care Medicine, Peking University First Hospital, Beijing, China; 2 Department of Critical Care Medicine, Xuanwu Hospital, Capital Medical University, Beijing, China; 3 Department of Clinical Epidemiology, Peking University First Hospital, Beijing, China; 4 Department of Anesthesiology, Peking University First Hospital, Beijing, China; 5 Department of Intensive Care Unit, Beijing Shijitan Hospital, Capital Medical University, Beijing, China; 6 Department of Geriatrics, Beijing Jishuitan Hospital, Capital Medical University, Beijing, China

**Keywords:** bloodstream infection, diagnosis, ICU, mNGS, perioperative

## Abstract

**Background:**

Bloodstream infections (BSI) in intensive care unit (ICU) patients are associated with high morbidity and mortality, necessitating rapid and accurate pathogen identification to guide early antimicrobial therapy. However, traditional blood culture (BC) is limited by the long turnaround time and low sensitivity. Metagenomic next-generation sequencing (mNGS) has been applied in infectious disease diagnostics, but its clinical utility for perioperative ICU patients with BSI requires further evaluation.

**Methods:**

This *post-hoc* analysis included 219 perioperative ICU patients (from a prospective, multi-center cohort, July 2020–June 2023) who underwent concurrent mNGS and BC testing. The study compared pathogen detection differences between the two methods, and evaluated the diagnostic value of mNGS for clinical BSI based on mNGS-assisted clinical diagnostic criteria. Additionally, the impact of mNGS findings on clinical antimicrobial management was assessed.

**Results:**

mNGS demonstrated a higher overall pathogen detection rate than BC in the 219 enrolled patients (25.1% vs. 9.6%, *p* < 0.001), with significant advantages in detecting Gram-negative bacteria (13.2% vs. 5.9%, *p* = 0.009), anaerobes (3.6% vs. 0.5%, *p* = 0.018), and fungi (6.4% vs. 0.9%, *p* = 0.002). Mixed-pathogen infections were identified in 20% of mNGS-positive clinical BSI cases, whereas BC-positive cases exclusively had single-pathogen infections. Ultimately, 64 patients (29.2%) were diagnosed with clinical BSIs. The sensitivity and specificity of the mNGS were 85.9% (95% CI: 74.5%–93.0%), and 80.6% (95% CI: 73.4%–86.4%), respectively, and the area under the receiver operating characteristic curve was 0.833 (95% CI: 0.772–0.894). The positive predictive value and negative predictive value were 64.7% (95% CI: 53.5%–74.6%) and 93.3% (95% CI: 87.3%–96.7%), respectively. Additionally, mNGS led to a positive impact in 56 patients (25.6%), manifested by the identification of new pathogens and guidance for targeted therapy, a negative impact in 11 patients (5.0%), and no clinical impact in 152 patients (69.4%).

**Conclusions:**

For perioperative ICU patients, mNGS demonstrated superior pathogen detection rates, broader microbial spectrum coverage, and enhanced polymicrobial infection detection capability versus BC. mNGS exhibited high diagnostic value for clinical BSI, with the potential to facilitate targeted antimicrobial therapy adjustments.

## Introduction

Bloodstream infections (BSI) represent one of the most severe infection types and account for 40% of sepsis or septic shock cases (Timsit et al., 2020). The incidence of BSI in hospitalized patients is 6.5 per 1,000 (Cui et al., 2022). Intensive care unit (ICU) patients exhibit a higher incidence of ICU-acquired BSI, reaching 5.2%, due to risk factors such as advanced age, multiple comorbidities, malignancies, immunosuppression, and malnutrition (Prowle et al., 2011; Seo et al., 2021). Furthermore, perioperative ICU patients are particularly susceptible to BSI, as their compromised physical and immune barriers increase the risk of microbial entry into the bloodstream (Wu et al., 2022). Rapid diagnosis and targeted antimicrobial therapy are crucial for BSI patients, particularly those complicated by sepsis or septic shock, as delayed or inappropriate treatment correlates with prolonged hospitalization, extended ICU stays, multidrug-resistant infections, and higher mortality (Tabah et al., 2012; Timsit et al., 2020).

Although blood culture (BC) remains the gold standard for diagnosing BSI, its clinical utility is limited by the prolonged turnaround time (3–5 days) and low sensitivity (30%–40% in sepsis), particularly in patients with prior antimicrobial exposure (Lamy et al., 2016; Schmitz et al., 2013). Contrastingly, plasma metagenomic next-generation sequencing (mNGS) identifies microorganisms through unbiased sequence analysis of circulating cell-free DNA (cfDNA) from plasma and has emerged as a promising diagnostic approach for critically ill patients (Brenner et al., 2025; Grumaz et al., 2016; Long et al., 2016). Studies reported mNGS positivity rates of 47%–66% in ICU/emergency patients with suspected BSI, exceeding concurrent BC results and providing additional pathogen information (Leitl et al., 2023; Liu et al., 2023; Zhou et al., 2023). However, mNGS results may include colonized or contaminant microorganisms alongside pathogens, necessitating the evaluation of its diagnostic value through alignment with clinical diagnoses (Blauwkamp et al., 2019; Simner et al., 2018). In this multicenter study, mNGS and BC results from perioperative ICU patients with suspected BSI were analyzed to compare pathogen detection yields. We evaluated the diagnostic value of mNGS for clinical BSI, and assessed the impact of mNGS results on clinical antimicrobial management.

## Methods

### Study population

This *post-hoc* analysis utilized data from the prospective, multicenter cohort study “Establishing a Prevention and Control System for BSI in Elderly Patients” (ChiCTR2100042168). From July 2020 to June 2023, the parent study enrolled 1,426 non-pregnant adults (≥18 years) with clinically suspected BSI across 10 tertiary hospitals in China. For the present analysis, patients were eligible if they met all of the following criteria: (1) both BC and mNGS were performed concurrently at the time of suspected BSI; (2) the patient was in the perioperative period, defined as the interval spanning preoperative evaluation, the intraoperative period, and the first 30 postoperative days; and (3) the patient was admitted to the ICU. Patients without paired BC/mNGS results or with incomplete key clinical data were excluded. Although the parent cohort included 10 hospitals, only four centers contributed patients who met all eligibility criteria for this *post-hoc* analysis. In total, 219 patients were included. Details of the participating centers, study period, and patient recruitment are provided in **Supplementary Table 1**. The study protocol received approval from the Peking University First Hospital Ethics Committee (2021KY211) and the respective ethics committees of all participating sites, with written informed consent obtained from patients or their legal representatives.

### Measurements

BC and blood mNGS samples were collected simultaneously when BSI was clinically suspected. The attending physician identified suspected BSI based on the following clinical manifestations: (1) systemic inflammatory response [fever (>38.3 °C or <36 °C), tachycardia (>90 bpm), tachypnea (>20 breaths per minute), or abnormal white blood cell count (>12 × 10^9^/L or <4 × 10^9^/L)], (2) infection-related symptoms [chills, profuse sweating, hypotension (systolic blood pressure < 90 mmHg or a drop > 40 mmHg from baseline), or clinical manifestations associated with potential infection sites], (3) supportive laboratory/imaging evidence (elevated C-reactive protein or procalcitonin levels, or microbiological/imaging evidence supporting the presence of an infectious focus).

### Blood culture collection and testing

BC were collected in two sets, obtained either via venipuncture or from a central venous catheter (CVC). For patients with a CVC indwelling time exceeding 48 hours and suspected catheter-related BSI, one set was drawn from the CVC and one set via peripheral venipuncture. In all other cases, both sets were collected by peripheral venipuncture. Each set comprised aerobic and anaerobic bottles, with 8–10 mL of blood inoculated per bottle. Samples were transported to the microbiology laboratories of the participating centers for incubation. Positive BCs underwent bacterial identification and antimicrobial susceptibility testing. Negative cultures were incubated for a minimum of 5 days.

### Blood mNGS collection and testing

For mNGS testing, blood samples (3–5 mL) were collected via venipuncture into EDTA anticoagulant tubes. Following collection, samples underwent centrifugation at 1,900 *g* for 10 minutes at 4 °C. The resulting plasma supernatant was then carefully aspirated and transferred to new 15-mL conical-bottom centrifuge tubes. A second centrifugation step was performed at 16,000 *g* for 10 minutes at 4 °C. The DNA was extracted by transferring 0.5 mL sample to a new 1.5-mL microcentrifuge tube, and the DNA was extracted using a MAPMI sample preparation kit (360120, CapitalBio Corporation, Beijing, China) according to the manufacturer’s recommendation.

DNA libraries were constructed through enzymatic DNA fragmentation (200–300 bp), end repair, the addition of adapters, and polymerase chain reaction (PCR) amplification. Following construction, the libraries were first assessed using an Agilent 2100 Bioanalyzer (Agilent Technologies, Santa Clara, CA, USA) to evaluate fragment size distribution and to exclude those with obvious abnormal peaks or adapter-dimer contamination. Quantitative PCR (qPCR) was subsequently performed on an Applied Biosystems 7500 Real-Time PCR System (Thermo Fisher Scientific, Waltham, MA, USA) with primers complementary to the adapter sequences, specifically quantifying adapter-ligated and amplifiable library molecules. Libraries meeting both the expected size distribution on the Bioanalyzer and a sufficient qPCR-determined concentration were considered qualified, and were then pooled and sequenced with an Ion PI chip on the BES4000 platform (CapitalBio Corporation).

For the bioinformatic analysis, the original sequencing data underwent quality control, and <50 bp, low-quality, and low-complexity reads were removed. The remaining high-quality sequencing data were mapped to the human reference genome GRCh38 and depleted for human host sequencing using Bowtie 2 v2.4.4.1. Subsequently, the non-human sequences were classified by simultaneous alignment to virus, bacteria, fungi, and parasite databases for annotation. All pathogenic genomic sequences were downloaded from the NCBI and PATRIC databases. The bacterial database contains 13,992 species, the fungal database contains 1659 species, the virus database contains 13,000 species, and the parasite database contains the genomic data of 287 pathogens. Suspected pathogens in clinical samples were evaluated by reviewing the data of different sample types from healthy individuals, and the relevant reference values were calculated, including hit read numbers and microorganism coverage. A water-only sample was processed in parallel as a negative control to monitor background contamination during sample preparation, library construction, and sequencing. Microorganisms detected in the negative control were treated as potential background contaminants and were used to contextualize detections in patient samples. The final pathogen detection report included a list of suspected pathogens, the hit read number, coverage degree, and confidence level of pathogenicity (high, medium or low; obtained from a comprehensive analysis of the hit read number, coverage degree, and quality control results). The process spanned approximately 24–48 h from DNA extraction to report issuance.

### Clinical data

Clinical data were extracted from electronic medical records, encompassing demographic characteristics, comorbidities, diagnoses, treatments, disease severity scores, surgical interventions, infection sources, and clinical outcomes. Sepsis and septic shock were classified according to the Sepsis-3 criteria. Sepsis was defined as suspected or documented infection associated with an acute increase in Sequential Organ Failure Assessment (SOFA) score of ≥2 points. Septic shock was defined as sepsis requiring vasopressor therapy to maintain a mean arterial pressure of ≥65 mmHg and a serum lactate level >2 mmol/L despite adequate fluid resuscitation (Singer et al., 2016). Culture results from potential infection sources (e.g., drainage fluid, respiratory secretions, urine, cerebrospinal fluid, surgical sites) were collected within a 7-day window centered on the onset of suspected BSI (± 7 days). Antimicrobial management decisions made by clinicians before and immediately after the availability of mNGS reports were systematically documented. Infection control status was assessed for the 7-day period following suspected BSI onset.

### Definitions

Classic contaminants identified in BC, such as coagulase-negative staphylococci, were evaluated for the possibility of being false positives based on the number of positive bottles, time to positivity, growth quantity, and clinical assessment. Cases confirmed as false positives were excluded from BC-positive analyzes. Detection of ≥1 microorganism by either mNGS or BC defined a positive result; absence of microorganisms defined a negative result. Paired BC and mNGS results were categorized as concordant (including both positive or negative agreement) or discordant. Positive concordance required ≥1 overlapping microorganism between BC and mNGS. Negative concordance was assigned if both methods yielded negative results. Discordance was assigned if all detected microorganisms differed between the two methods. Blood samples underwent additional viral diagnostic testing (real-time qPCR) only when viral DNA detected by mNGS was deemed clinically relevant.

**Figure 1:**
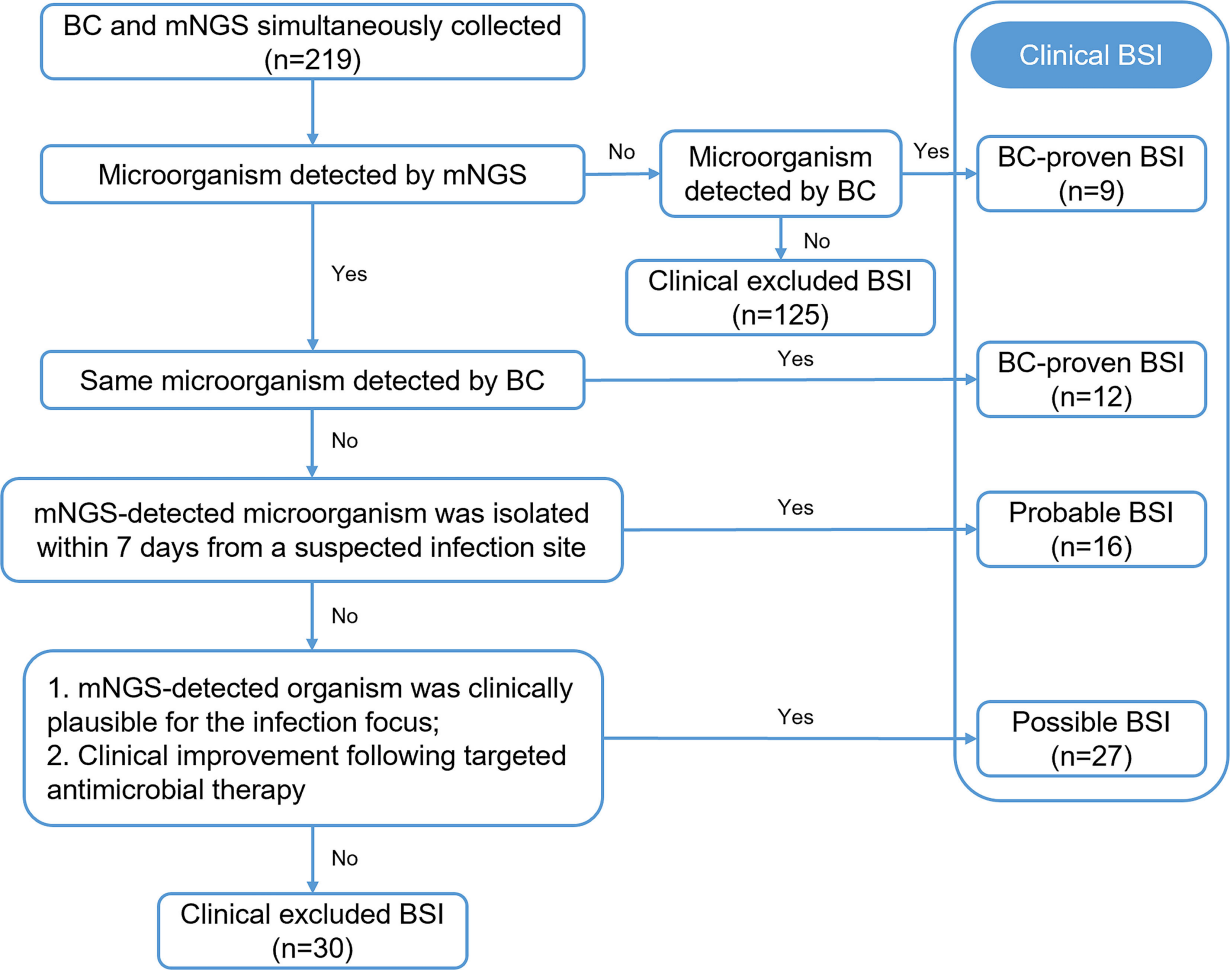
Flowchart of pathogenic microorganism identification and results analysis.

“BC-proven BSI” was defined as a positive BC result accompanied by compatible systemic manifestations of infection, and was classified as clinical BSI. For BC-negative cases, two additional clinical BSI categories were established based on an integrated assessment of mNGS results, microbiological analyzes from suspected infection sites, and clinical response: (1) “probable BSI” (positive mNGS results corroborated by microbiological identification of the same microorganism from a suspected infection site within 7 days), and (2) “possible BSI” (positive mNGS with negative or discordant microbiological culture results from infection sites, but the mNGS-detected microorganism was clinically plausible for the infection focus, accompanied by clinical improvement following targeted antimicrobial therapy). “Non-clinical BSI” encompassed cases that did not meet the diagnostic criteria for BC-proven, probable, or possible BSI (Nguyen et al., 2019; Wu et al., 2022) (**Figure 1**). Two researchers forming a review panel assessed each patient’s diagnosis of clinical BSI independently. Differences between the two researchers should be thoroughly discussed with the clinical physician to reach a final agreement.

Clinical impact was assessed based on the final diagnosis of clinical BSI, evaluating the decisions made by the clinicians after interpreting the mNGS results. “Positive clinical impact” was defined as mNGS exclusively or earlier identifying the BSI pathogen, guiding clinicians to: (1) initiate new antimicrobial therapy, (2) escalate existing therapy, or (3) maintain the current therapy. “Negative clinical impact” was defined as mNGS: (1) failing to detect pathogens later identified by BC, or (2) leading to unnecessary therapies due to false-positive results. “No clinical impact” was defined as clinicians not adjusting antimicrobial therapy due to: (1) negative mNGS results, or (2) the assessment that detected microorganisms were clinically irrelevant (Hogan et al., 2021; Niles et al., 2022).

### Statistical analysis

Continuous variables with normal distribution are presented as mean ± standard deviation and compared using Student’s *t*-test. Non-normally distributed continuous variables are expressed as median [interquartile range (IQR)] and analyzed with the Mann-Whitney *U* test. Categorical variables are reported as frequencies (percentages), with between-group differences evaluated by χ² test or Fisher’s exact test as appropriate. For significant differences in multinomial categorical variables, *post-hoc* pairwise comparisons were adjusted using Bonferroni correction. Agreement between BC and mNGS results was assessed with McNemar’s χ² test. All tests were two-sided, with *p* < 0.05 considered statistically significant. Diagnostic performance of mNGS for clinical BSI was evaluated by calculating sensitivity, specificity, positive predictive value (PPV), and negative predictive value (NPV) using standard 2×2 contingency tables. Receiver operating characteristic (ROC) analysis was performed, with the area under the curve (AUC) quantifying overall diagnostic accuracy.

## Results

### Study population

The cohort (n=219) had a median age of 67 years (IQR 55–75) with male predominance (68.9%, 151/219). Sepsis was diagnosed in 93.6% (205/219) of patients, including septic shock in 43.4% (95/219). Most patients (87.7%, 192/219) received antimicrobial therapy within 24 hours preceding BC/mNGS testing. High surgical risk was evident, with 86.3% (189/219) classified as ASA physical status III-V and 43.8% (96/219) undergoing emergency procedures. Abdominopelvic surgeries constituted the majority (51.6%, 113/219), followed by neurosurgical interventions (24.2%, 53/219). Abdominal sources accounted for 45.7% (100/219) of primary infections, while pulmonary sources represented 23.3% (51/219).

Stratification according to clinical BSI diagnosis revealed significant inter-group disparities. The clinical BSI group had significantly older age, higher autoimmune disease prevalence, elevated disease severity scores, increased septic shock incidence, and a greater proportion of emergency surgical interventions than the non-clinical BSI group. Significant differences in infection sources were also observed between groups (pulmonary vs. none; skin and soft tissue vs. none; catheter-related vs. none) (all *p* < 0.05) (**Table 1**).

**Table 1:** Patient characteristics.

Characteristics	Total (n=219)	Clinical BSI (n=64)	Non-clinical BSI (n=155)	*p*
Age (years)	67(55,75)	70(62,77)	66(53,74)	0.039
Male sex	151(68.9)	43(67.2)	108(69.7)	0.717
Comorbid conditions				
Hypertension	108(49.3)	34(53.1)	74(47.7)	0.469
Coronary heart disease	36(16.4)	12(18.8)	24(15.5)	0.553
Chronic obstructive pulmonary disease	4(1.8)	2(3.1)	2(1.3)	0.582
Neurologic disorder	34(15.5)	13(20.3)	21(13.5)	0.209
Diabetes mellites	47(21.5)	12(18.8)	35(22.6)	0.53
Chronic kidney disease	12(5.5)	4(6.3)	8(5.2)	0.75
Autoimmune disease	8(3.7)	6(9.4)	2(1.3)	0.009
Malignant tumors	113(51.6)	33(51.6)	80(51.6)	0.995
Disease severity scores				
SOFA score	7(4,9)	8(6,10)	6(4,9)	0.015
APACHE II score	13(9,19)	15(11,20)	12(7,18)	0.011
Diagnoses				
Sepsis	205(93.6)	60(93.8)	145(93.5)	1
Septic shock	95(43.4)	35(54.7)	60(38.7)	0.03
Treatment				
Antimicrobial treatment 24h before mNGS/BC	192(87.7)	53(82.8)	139(89.7)	0.16
Prophylactic perioperative application	46(21.0)	10(15.6)	36(23.2)	0.209
Therapeutic application	146(66.7)	43(67.2)	103(66.5)	0.916
Ventilator	172(78.5)	54(84.4)	118(76.1)	0.176
Surgical intervention				
ASA classification				0.232
I-II	30(13.7)	6(9.4)	24(15.5)	
III-IV	189(86.3)	58(90.6)	131(84.5)	
Type of surgery				0.947
Abdominal and pelvic surgery	113(51.6)	32(50.0)	81(52.3)	
Neurosurgery	53(24.2)	15(23.4)	38(24.5)	
Thoracic surgery	17(7.8)	5(7.8)	12(7.7)	
Orthopedic surgery	16(7.3)	6(9.4)	10(6.5)	
Others ^a^	20(9.1)	6(9.4)	14(9.0)	
Open surgery ^b^	187(85.4)	55(85.9)	132(85.2)	0.882
Emergency surgery	96(43.8)	35(54.7)	61(39.4)	0.038
Duration of surgery (min)	162(83,286)	161(102,239)	163(83,297)	0.316
Interval from surgery to mNGS/BC sampling (d)	2(0,8)	5(0,10)	2(0,6)	0.046
Source of infection				<0.001
Intra-abdominal infection	100(45.7^*^)	28(43.8)	72(46.5^*^)	0.635
Gastric/duodenal perforation	7(3.2)	2(3.1)	5(3.2)	
Small intestine necrosis/perforation	20(9.1)	4(6.3)	16(10.3)	
Colorectal perforation	16(7.3)	6(9.4)	10(6.5)	
Acute biliary infection	11(5.0)	5(7.8)	6(3.9)	
Liver abscess	3(1.4)	1(1.6)	2(1.3)	
Infected necrotizing pancreatitis	14(6.4)	2(3.1)	12(7.7)	
Postoperative anastomotic leak	20(9.1)	6(9.4)	14(9.0)	
Postoperative biliary leak	4(1.8)	2(3.1)	2(1.3)	
Postoperative pancreatic leak	3(1.4)	0(0)	3(1.9)	
Bladder rupture/postoperative bladder leak	2(0.9)	0(0)	2(1.3)	
Pulmonary infection	51(23.3)	19(29.7)	32(20.6)	
Intracranial infection	14(6.4)	3(4.7)	11(7.1)	
Skin and soft tissue infection	12(5.5)	7(10.9^*^)	5(3.2)	0.015
Gangrene of the extremities	6(2.7)	1(1.6)	5(3.2)	
Surgical site infection	3(1.4)	3(4.7)	0(0)	
Auricular abscess	3(1.4)	3(4.7)	0(0)	
Urinary system infection	11(5.0)	2(3.1)	9(5.8)	
Mediastinal infection	7(3.2)	2(3.1)	5(3.2)	
Catheter-associated BSI	3(1.4)	3(4.7)	0(0)	
None	21(9.6)	0(0)	21(13.5)	–
Postoperative inflammatory response	13(5.9)	0(0)	13(8.4)	
Central fever	5(2.3)	0(0)	5(3.2)	
Transfusion reaction	2(0.9)	0(0)	2(1.3)	
Neoplastic fever	1(0.5)	0(0)	1(0.6)	
Clinical outcome				
28-day mortality	44(20.1)	12(18.8)	32(20.6)	0.491

Data are presented as median (interquartile range) or number of patients (percentage).

^*^ Percentages do not sum to total due to rounding.

^a^ including thyroid surgery, peripheral vascular surgery, and endoscopic procedures (endoscopic retrograde cholangiopancreatography, bronchoscopy, ureteroscopy, etc.).

^b^ open or laparoscopic surgery.

APACHE II, acute physiology and chronic health evaluation II; ASA, American society of anesthesiologists; BC, blood culture; BSI, bloodstream infection; mNGS, metagenomic next-generation sequencing; SOFA, sequential organ failure assessment.

### mNGS microbial detection results

**Figure 2:**
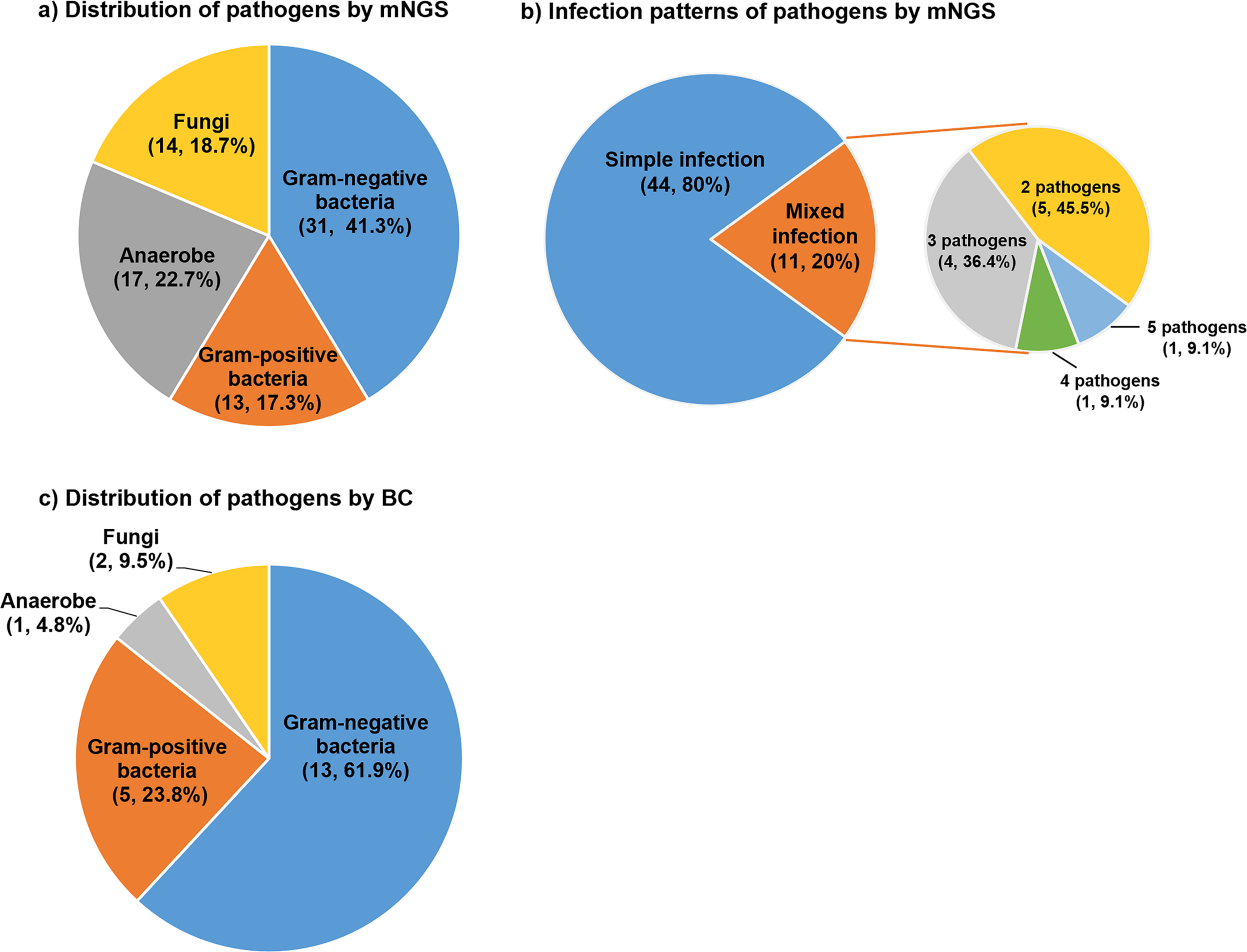
Distribution of pathogens detected by mNGS and BC **(a)** Distribution of pathogens detected by mNGS; **(b)** Infection patterns of pathogens detected by mNGS; **(c)** Distribution of pathogens detected by BC.

**Figure 3:**
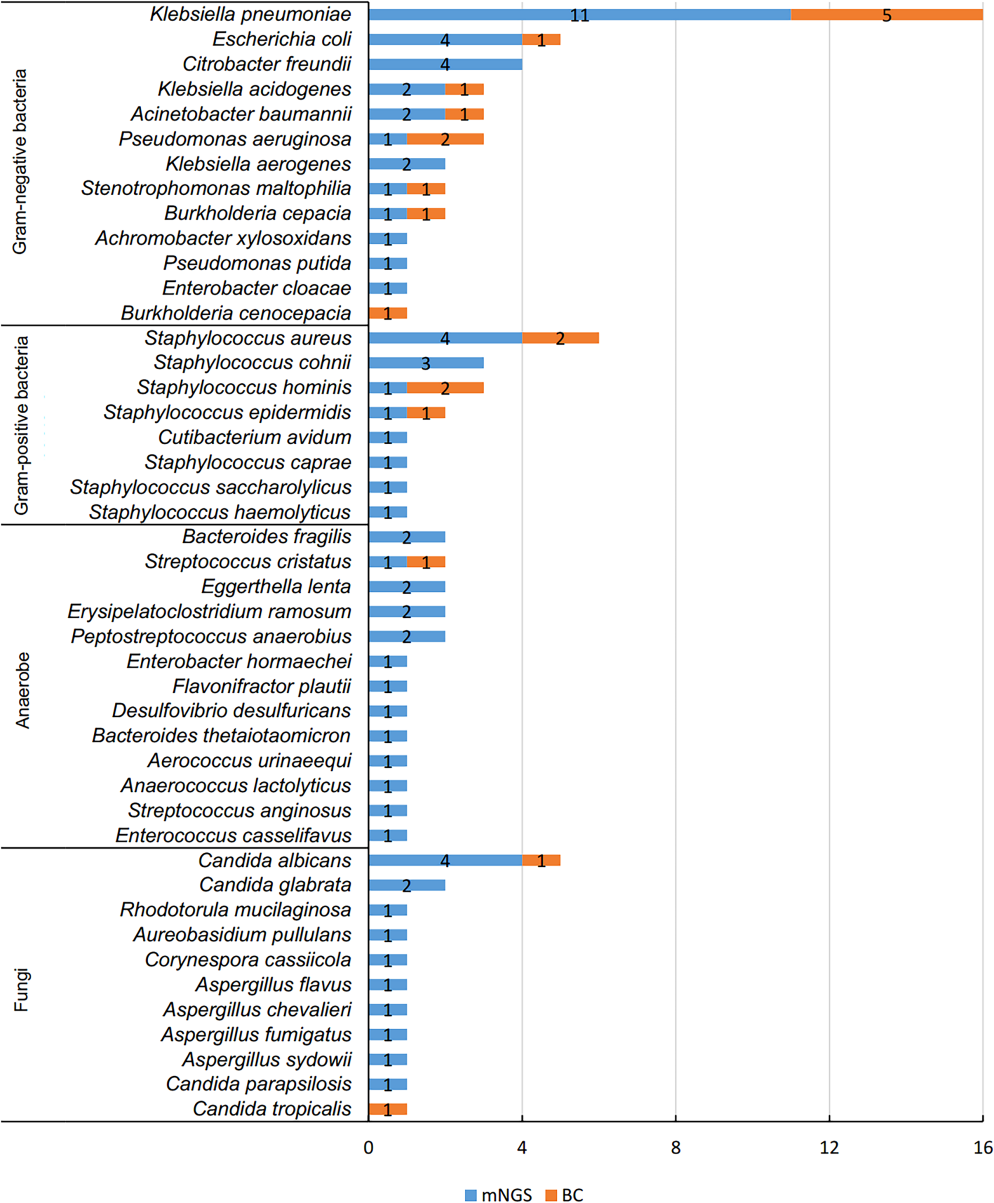
The specific pathogens detected by mNGS and BC.

In the distribution of detected microorganisms, anaerobes were listed separately because of their clinical relevance in perioperative infections; accordingly, the Gram-negative and Gram-positive bacterial categories refer to non-anaerobic bacteria. mNGS yielded positive results for 106 of 219 cases (48.4%) and identified 157 microorganisms: 92 bacteria (35 Gram-negative, 30 anaerobic, 27 Gram-positive), 42 viruses, and 23 fungi. Among the mNGS-positive patients, 55 (51.9%, 55/106) were diagnosed with clinical BSI (defined as BC-proven, probable, or possible BSI), involving 75 pathogenic microorganisms. These comprised 31 Gram-negative bacteria (41.3%), 17 anaerobes (22.7%), 14 fungi (18.7%), and 13 Gram-positive bacteria (17.3%). Among the 17 anaerobes, 10 were Gram-positive, 5 were Gram-negative, and 2 were Gram-variable. No viruses associated with BSI were identified (**Figure 2a**). Polymicrobial infections (≥2 pathogens) occurred in 20% (11/55) of clinical BSI cases, with pathogen counts per case as follows: 1 case (5 pathogens), 1 case (4 pathogens), 4 cases (3 pathogens), and 5 cases (2 pathogens) (**Figure 2b**). Gram-negative bacteria exhibited the highest detection rate and were predominantly linked to intra-abdominal and pulmonary infections. Anaerobes, exclusively of intestinal origin, were associated with intra-abdominal infections. Fungi were mainly associated with abdominal and pulmonary sources, while Gram-positive bacteria primarily correlated with pulmonary and abdominal infections. The most frequently detected pathogens were *Klebsiella pneumoniae* (n=11), *Escherichia coli* (n=4), *Citrobacter freundii* (n=4), *Staphylococcus aureus* (n=4), and *Candida albicans* (n=4) (**Figure 3**).

### Blood culture microbial detection results

After exclusion of four contaminants, BC yielded clinically significant positive results in 21 of 219 cases (9.6%). The excluded contaminants comprised two coagulase-negative staphylococci, one *Bacillus* spp., and one *Candida* spp. The clinically significant positive BC findings represented monomicrobial infections: 13 Gram-negative bacteria (61.9%), 5 Gram-positive bacteria (23.8%), 2 fungi (9.5%), and 1 anaerobic bacterium (4.8%; Gram-positive). *K. pneumoniae* was the most frequently detected pathogen (n = 5) (**Figures 2c**, **3**).

### Comparison of mNGS and blood culture

mNGS showed a higher diagnostic yield for clinical BSI than BC (25.1% vs. 9.6%, *p* < 0.001). Specifically, mNGS identified more cases involving Gram-negative bacteria (29 vs. 13, *p* = 0.009), anaerobic bacteria (8 vs. 1, *p* = 0.037), and fungi (14 vs. 2, *p* = 0.002) (**Supplementary Figure 1**). Among fungal pathogens, BC only detected yeast species, whereas mNGS identified both yeast and filamentous fungi (including four *Aspergillus* cases). Combining mNGS with BC significantly increased the positive detection rate for clinical BSI from 9.6% (BC alone) to 29.2% (*p* < 0.001).

Regarding the impact of prior antimicrobial treatment on pathogen detection: Among 192 patients receiving antimicrobials within 24h before specimen collection, mNGS demonstrated significantly higher positivity rates for clinical BSI compared to BC [22.9% (44/192) vs. 8.9% (17/192); *p* < 0.001]. A similar pattern was observed in the 27 patients without recent antimicrobial therapy [40.7% (11/27) vs. 14.8% (4/27), *p* = 0.033].

Comparison of the mNGS results (excluding viruses) with BC revealed concordance for 137 cases (62.6%): 12 cases with positive concordance (5.5%) and 125 cases with negative concordance (57.1%). Eighty-two cases had discordant results (37.4%): 9 cases with positive BC only (4.1%) and 73 cases with positive mNGS only (33.3%). McNemar’s χ^2^ test indicated that the two methods had significantly different diagnostic performance (*p* < 0.001).

Among the 12 patients with positive concordance, 6 (50%) had not received antimicrobials prior to blood sampling, 7 (58.3%) presented with septic shock, and pathogens were detected in at least half of the BC bottles (3 of 4 or 2 of 4) in all cases (100%). Of the 125 patients with negative concordance, most (86.4%) were considered to have localized infections, primarily involving the abdomen or lungs, while a subset (13.6%) was likely in a non-infectious state, with clinical manifestations attributed to surgical stress, central fever, transfusion reactions, or tumor-related fever.

Among nine BC-positive-only patients, five were Gram-negative, three were Gram-positive, and one was fungus. The Gram-negative organisms not detected by mNGS included three *Pseudomonas aeruginosa*, one *Acinetobacter baumannii*, and one *K. pneumoniae*, and caused infections from intra-abdominal, pulmonary, and skin/soft tissue sources. Among the three Gram-positive bacteremia cases confirmed by BC, two involved coagulase-negative staphylococci that were associated with central venous catheter-related BSI, while one was caused by *S. aureus* originating from pulmonary infection. The fungal BSI case was identified as *Candida tropicalis* secondary to a urinary tract infection. Only 22% (2/9) of patients had pathogens detected in at least half of BC bottles: one with *P. aeruginosa* bacteremia secondary to pneumonia and one with *K. pneumoniae* bacteremia from a deep incisional surgical site infection. The remaining seven patients (78%) with only 1/4 positive BC bottles demonstrated clinical manifestations corroborated by either microbiological confirmation at primary infection sites or, for the two catheter-associated BSI, clinical resolution following catheter removal and targeted antibiotic therapy. All nine patients (100%) had received antimicrobial therapy prior to blood collection.

Among the 73 mNGS-positive-only patients (excluding viral detections), 16 met criteria for probable BSI, with culture-confirmation of identical microorganisms at localized infection sites: intra-abdominal (n=9), respiratory (n=4), urinary tract (n=1), intracranial (n=1), and skin/soft tissue (n=1). Twenty-seven patients met criteria for possible BSI, where mNGS identified clinically plausible pathogens corresponding to primary infection sites: intra-abdominal (n=10), respiratory (n=10), skin/soft tissue (n=5), intracranial (n=1), and mediastinal (n=1). Targeted antimicrobial therapy directed against these identified pathogens resulted in observable clinical improvement (**Figure 1**).

Sixteen mNGS-positive-only patients involved enteric anaerobic bacteria, with eight (50%, 8/16) classified as possible BSI [25 strains detected, among which 16 (64%) were BSI-linked]. The result interpretation considered the cfDNA detection levels and referenced the previously reported pathogenicity of these microorganisms. Separately, skin/environmental microorganisms were detected in 25 cases, predominated by Gram-positive bacteria (coagulase-negative staphylococci), alongside fewer Gram-negative species and non-tuberculous mycobacteria. Eight of these 25 patients (32%) were classified as possible BSI [27 strains identified, among which 8 (29.6%) were deemed BSI-related], with primary infections involving the lungs (6 case), surgical site (1 case), and gastrointestinal tract (1 case). Diagnostic assessments synthesized cfDNA concentrations, microbial pathogenicity, and adherence to site-of-origin infection criteria.

### Diagnostic performance of mNGS for clinical BSI

**Figure 4:**
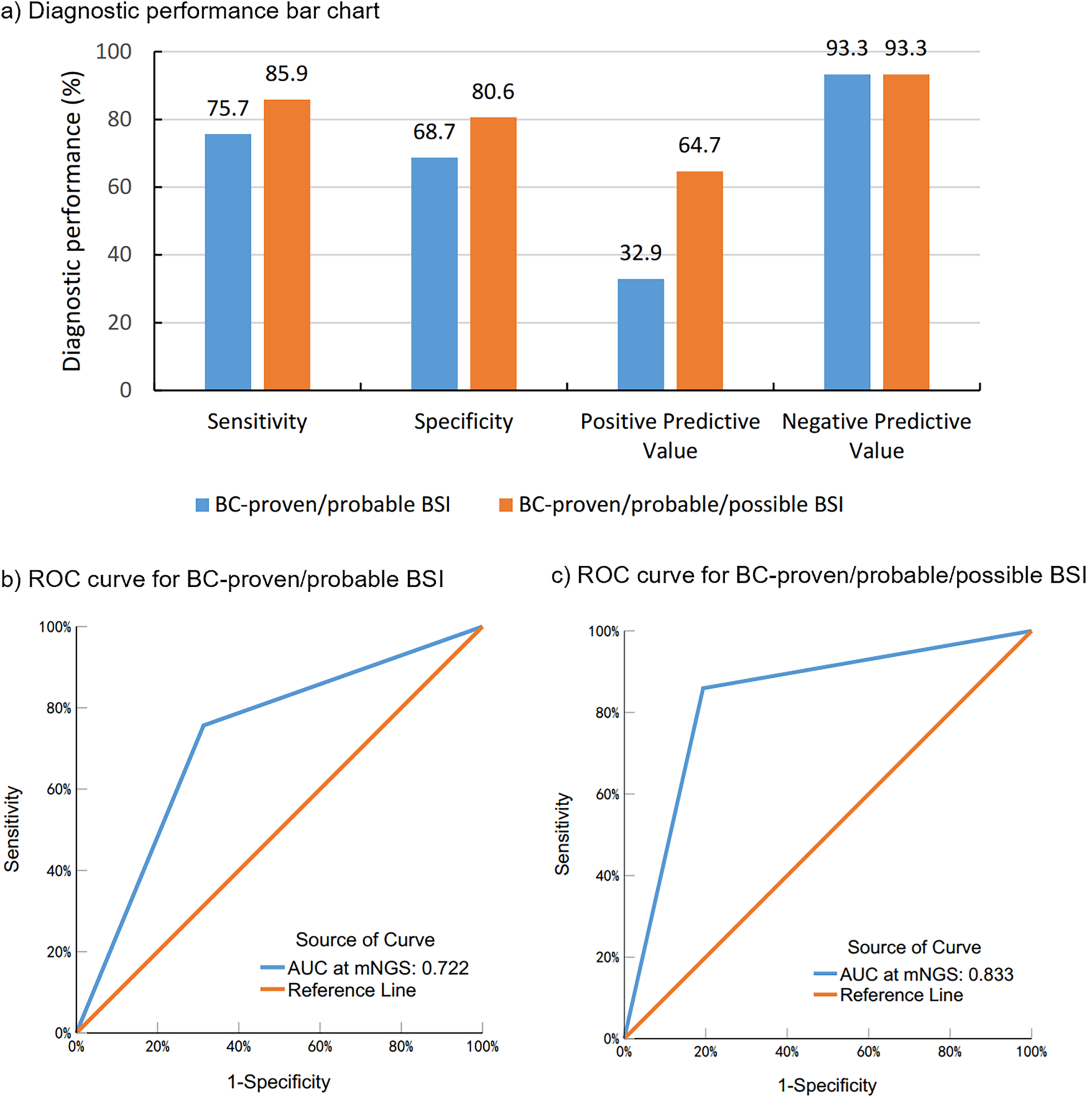
Diagnostic performance of mNGS under two clinical BSI definitions **(a)** Bar chart showing the sensitivity, specificity, PPV, and NPV of mNGS for diagnosing clinical BSI under two definitions: BC-proven/probable BSI and BC-proven/probable/possible BSI; **(b)** ROC curve of mNGS for the diagnosis of BC-proven/probable BSI (AUC = 0.722); **(c)** ROC curve of mNGS for the diagnosis of BC-proven/probable/possible BSI (AUC = 0.833).

When microbiological tests alone defined true-positive mNGS results in BC-negative cases, 37 patients were classified as clinical BSI (including BC-proven and probable BSI). Under this definition, mNGS demonstrated a sensitivity of 75.7% (95% CI: 58.4–87.6%) and specificity of 68.7% (95% CI: 61.3–75.2%), with an AUC of 0.722 (95% CI: 0.632–0.811). When both microbiological and clinical criteria defined true-positive mNGS results in BC-negative cases, clinical BSI cases increased to 64 (including BC-proven, probable, and possible BSI). This expanded definition yielded improved mNGS performance: sensitivity 85.9% (95% CI: 74.5–93.0%), specificity 80.6% (95% CI: 73.4–86.4%), and AUC 0.833 (95% CI: 0.772–0.894) (**Figure 4**; **Supplementary Table 2**).

### Impact of mNGS results on clinical management

Among 219 patients, mNGS positively influenced clinical management in 25.6% (56/219), through new BSI diagnoses enabling targeted antimicrobial therapy. This included exclusive pathogen detection by mNGS in 45 cases and earlier identification than BC in 12 cases, with one case overlapping between these categories. Corresponding therapeutic modifications predominantly involved: initiation of antifungal therapy (8 cases) and anti-Gram-positive antimicrobials (6 cases), along with escalation of anti-Gram-negative regimens (7 cases). Conversely, mNGS adversely affected management in 5.0% (11/219): nine cases maintained inappropriate therapy due to undetected BC-confirmed pathogens, while two received unnecessary glycopeptides for misinterpreted coagulase-negative staphylococci (one post-neurosurgery without infection; one with *E. coli* intra-abdominal infection without secondary BSI). mNGS showed no clinical impact in 69.4% (152/219), attributed to negative results (125 cases) and clinicians’ judgment of clinical irrelevance (27 cases). Viral results were excluded from impact analysis due to absent BSI association (**Table 2**).

**Table 2:** Clinical impact of mNGS.

Clinical impact	Number of cases (%)
Positive impact	56 (25.6) ^*^
Exclusive diagnosis independent of BC	45 (20.5)
Earlier diagnosis later confirmed by BC	12 (5.5)
Initiation of antimicrobials	14 (6.4)
Targeting fungi	8 (3.7)
Targeting Gram-positive bacteria	6 (2.7)
Escalation of antimicrobials	10 (4.6)
Targeting Gram-negative bacteria	7 (3.2)
Targeting fungi	2 (0.9)
Targeting anaerobic bacteria	1 (0.5)
Maintenance of current antimicrobials based on new diagnosis	32 (14.6)
Negative impact	11 (5.0)
Missed diagnosis confirmed by BC	9 (4.1)
Unnecessary therapy led by false-positive results	2 (0.9)
No impact	152 (69.4)
No change in antimicrobials based on negative results	125 (57.1)
No change in antimicrobials based on deemed clinical irrelevance of positive results	27 (12.3)

^*^ There is overlap in cases in the positive impact category. Each case was only counted once for overall positive impact.

BC, blood culture; mNGS, metagenomic next-generation sequencing.

## Discussion

While prior studies (Leitl et al., 2023; Liu et al., 2023; Zhou et al., 2023) have established the superior diagnostic performance of mNGS over BC for BSI in general ICU populations, its diagnostic utility in perioperative ICU patients remained undefined. In this multicenter diagnostic study, mNGS showed a higher pathogen detection rate than BC in critically ill perioperative patients, most of whom had received antimicrobial therapy before sampling. mNGS improved the detection of Gram-negative bacteria, anaerobes, fungi, and polymicrobial infections. It also showed good diagnostic performance for clinical BSI and provided useful information for antimicrobial management.

The overall mNGS positivity rate was 48.4%, which was slightly lower than that reported in previous ICU studies of suspected BSI (52.0% and 68.5%) (Leitl et al., 2023; Liu et al., 2023). This difference may be related to the higher rate of prior antimicrobial exposure in our cohort. Gram-negative bacteria were the predominant pathogens, consistent with previous ICU BSI studies (Tabah et al., 2012). Anaerobic bacteria were detected more frequently than in general ICU populations. This finding may reflect the perioperative characteristics of our cohort. Abdominopelvic and gastrointestinal surgery can disrupt mucosal barriers and promote translocation of gut anaerobes into the bloodstream. Postoperative complications may also create hypoxic environments that favor anaerobic growth (Cobo et al., 2023; Saito et al., 2003). Gram-positive bacteremia was mainly associated with pulmonary infection, catheter-related infection, and surgical site infection, which are common infection sources in perioperative ICU patients (Barnes et al., 2019; Timsit et al., 2020).

Compared to BC, mNGS demonstrated a higher pathogen detection rate (25.1% vs. 9.6%), attributable to their fundamental methodological differences. Unlike BC, which requires viable microbial proliferation, mNGS detects pathogens via nucleic acid identification independent of viability. BC’s lower sensitivity for anaerobic bacteria and fungi may be due to their stringent culture requirements or slower growth (Hu et al., 2021). Aspergillus species were identified only by mNGS, which is consistent with previous reports and supports the complementary value of mNGS for invasive fungal infection diagnosis (Duan et al., 2021; Liu et al., 2023). mNGS also identified polymicrobial infections in 15.1% of mNGS-positive BSI cases, whereas BC detected only monomicrobial infections. This discrepancy likely stems from mNGS’s unbiased capture of plasma cfDNA from multiple pathogens, whereas the inherent biological competition of BC allows rapidly proliferating microorganisms to suppress co-infecting strains (Lee et al., 2022; Sun et al., 2022).

Prior antimicrobial exposure was common in this cohort (87.7%). Antimicrobial therapy before sampling reduced the detection rates of both mNGS and BC. However, mNGS still maintained a higher positivity rate than BC after antimicrobial exposure, consistent with previous evidence (Jing et al., 2021). These findings demonstrate that for critically ill perioperative patients—who frequently receive antimicrobial prophylaxis for surgery or therapeutic regimens due to clinical deterioration in general wards, making pre-antimicrobial blood collection challenging—mNGS sustains persistently high diagnostic sensitivity for BSI.

Discordant results between mNGS and BC require careful interpretation. In BC-positive-only cases, mNGS false negativity may be related to low pathogen burden, sequence counts below reporting thresholds, high levels of host cfDNA, or degradation of microbial cfDNA during sample processing (Fida et al., 2021; Leitl et al., 2023; Niles et al., 2023; Zhou et al., 2023). Most of these cases had only one positive BC bottle and had received antimicrobials before sampling, suggesting low microbial load. These findings support the continued combined use of mNGS and BC for BSI diagnosis.

One-third of patients had positive mNGS results but negative BC results. These results may represent several clinical scenarios. First, they may indicate persistent BSI in which antimicrobial therapy suppresses microbial growth in culture. Second, they may reflect intermittent or transient bacteremia from focal infections, such as pneumonia, liver abscess, or intra-abdominal infection. Third, they may represent detection of colonizing organisms or contaminants (Nguyen et al., 2019; Wu et al., 2022).Such organisms were generally considered clinically irrelevant, as they lacked support from BC or site-specific microbiology, were inconsistent with the clinical presentation, exhibited low abundance, and were listed in background databases. However, they may be clinically relevant in selected settings, such as immunosuppression, or high-burden detection (Hall and Lyman, 2006; Miller et al., 2018). We therefore interpreted mNGS-positive-only results according to predefined clinical BSI diagnostic criteria, whereby cases meeting the definitions of probable or possible BSI were considered more likely to represent true BSI. Microbial cfDNA concentration was also taken into account, because lower concentrations are more common in asymptomatic individuals and in organisms considered unlikely to be the cause of infection (Blauwkamp et al., 2019). Nevertheless, cfDNA concentration was not used as a standalone diagnostic threshold. Although quantitative or semi-quantitative microbiological criteria are incorporated into guidelines for selected infections, such as urinary tract infections and catheter-related BSIs, standardized quantitative thresholds are lacking for many other body sites and for plasma mNGS abundance in suspected BSI (Hooton et al., 2010; Mermel et al., 2009). As mNGS measures microbial cfDNA rather than viable colony-forming units, its abundance data should be interpreted together with clinical diagnostic criteria and other supporting evidence.

Because many patients in this study underwent abdominal surgery or had intra-abdominal infections, intestinal anaerobes were frequently detected by mNGS. Anaerobes are recognized causes of bacteremia, particularly in intra-abdominal and surgical infections, but they are often difficult to isolate using conventional culture methods (Bilen et al., 2018). Therefore, microbiological confirmation is often lacking, and mNGS-only detection of these organisms should be interpreted cautiously. Some gut commensals, including *Akkermansia muciniphila*, *Lactobacillus* spp., and *Bifidobacterium* spp., are generally regarded as mucosal commensals or beneficial microorganisms, but they have also been reported to cause invasive infections under specific high-risk conditions, such as immunosuppression or critical illness (Cani et al., 2022; Song et al., 2022; Salminen et al., 2004; Esaiassen et al., 2017). Thus, mNGS-detected intestinal anaerobes should be interpreted in the clinical context, especially in perioperative patients and those with intra-abdominal infections.

Among patients with suspected BSI, 29.2% were finally diagnosed with clinical BSI. These patients had greater disease severity and a higher incidence of septic shock. This finding highlights the need for accurate and timely etiological diagnosis in perioperative ICU patients. mNGS showed high sensitivity and acceptable specificity for clinical BSI diagnosis, with an AUC of 0.833. These results suggest that mNGS is a useful adjunctive diagnostic tool in this population. Its higher sensitivity may be especially valuable when BC positivity is reduced by perioperative prophylaxis or empirical antimicrobial therapy.

mNGS also influenced antimicrobial management. In this study, plasma mNGS had a positive clinical impact in approximately one-quarter of patients, which was higher than rates reported in some previous studies (Hogan et al., 2021; Niles et al., 2022). The main benefits were pathogen identification and targeted treatment adjustment. mNGS supported initiation of antifungal or anti-Gram-positive therapy and escalation of treatment for Gram-negative infections. This is important because empirical therapy in critically ill perioperative patients often focuses on Gram-negative coverage, while timely treatment for fungi, resistant Gram-positive bacteria, or resistant Gram-negative organisms requires more specific evidence (De Pascale et al., 2022; Evans et al., 2021). By providing earlier pathogen information, mNGS may help shorten the time to targeted therapy compared with conventional culture and serological testing (Timsit et al., 2019). Negative clinical impacts were mainly caused by mNGS false-negative results identified by positive BC, again supporting the combined use of both methods.

Several caveats regarding mNGS interpretation should be acknowledged. First, mNGS does not provide direct antimicrobial susceptibility testing (AST) and cannot replace CLSI/EUCAST-standardized culture-based AST for guiding antimicrobial decisions (Morrissey and Patel, 2025). Resistance gene detection may provide partial guidance, but it depends on curated databases and validated pipelines and requires further clinical validation (Alcock et al., 2023). Second, mNGS cannot always reliably differentiate true pathogens from colonizers or contaminants. Skin commensals, environmental organisms, and gut anaerobes of uncertain clinical significance may lead to overtreatment if evaluated without clinical context. Therefore, ICU clinicians should interpret positive mNGS results prudently, combining corroborating evidence from BCs and site-specific microbiological tests with clinical information.

This study has several limitations. First, as a *post-hoc* analysis of a prospective cohort, patient selection based on specific criteria may have introduced potential selection bias and limited generalizability. Second, enrollment was uneven across centers, with most patients recruited from two sites. This imbalance may reflect real-world differences in testing availability and patient eligibility, but it may also limit the representativeness of the multicenter cohort. Third, the subgroup analysis evaluating the impact of prior antimicrobial exposure was limited by the small number of antimicrobial-unexposed patients. Although mNGS showed significantly higher detection rates than BC, this finding should be validated in larger cohorts of treatment-naïve critically ill patients. Fourth, the interpretation of the blood mNGS results mainly relied on study-defined criteria and expert panel adjudication, reflecting the current absence of standardized diagnostic criteria. Nevertheless, the proposed diagnostic framework may nevertheless inform future standardization efforts.

## Conclusions

The present study demonstrates the advantage of mNGS for identifying pathogens in perioperative ICU patients with suspected BSI, particularly those with a high rate of prior antimicrobial exposure. Compared with BC, mNGS has a higher positivity rate, wider microbial detection range, and enhanced polymicrobial infection detection capability. Furthermore, mNGS shows good diagnostic performance for clinical BSI and facilitates targeted antimicrobial therapy adjustments.

## Data Availability

The raw data supporting the conclusions of this article will be made available by the authors, without undue reservation.
