# Supplementary material for: Performance of metagenomic next-generation sequencing for bloodstream infections in perioperative critically ill patients- a post-hoc analysis of a prospective, multi-center cohort study
Source: Front Cell Infect Microbiol. 2026 Jun 15;16:1814969. doi: 10.3389/fcimb.2026.1814969 (PMC13311091; doi:10.3389/fcimb.2026.1814969)
Supplement: Supplementary file 1 [file Table1.docx]

**Supplementary Materials for**

**Performance of metagenomic next-generation sequencing for bloodstream infections in perioperative critically ill patients- A post-hoc analysis of a prospective, multi-center cohort study**

Qiao Qin, Ya-Chan Ning, Sai-Nan Zhu, Jia-Hui Ma, Wei Chen, Wei Tian, Chun-Mei Wang^*^, Ying-Feng Wu^*^, Shuang-Ling Li^*^

***Corresponding authors:** Chun-Mei Wang, drwangchunmei@sina.com; Ying-Feng Wu, xwhvs@163.com; Shuang-Ling Li, lishuangling888@hotmail.com

**This PDF file includes:**

**Supplemental Table**

Supplemental Table 1

Supplemental Table 2

**Supplemental Figure Legends**

**Supplemental Figures**

Supplemental Figure 1

**Supplemental Table**

**Supplemental Table 1** Recruitment information for the present post-hoc analysis

| Center | Hospital name | City | Study period | No. of included patients |
| --- | --- | --- | --- | --- |
| 1 | Peking University First Hospital | Beijing | Jun 2021-May 2022 | 131 |
| 2 | Xuanwu Hospital | Beijing | Jun 2021-May 2022 | 80 |
| 3 | Beijing Shijitan Hospital | Beijing | Jun 2021-May 2022 | 6 |
| 4 | Beijing Jishuitan Hospital | Beijing | Jun 2021-May 2022 | 2 |

**Supplemental Table 2** Evaluation of the diagnostic performance of mNGS for clinical BSI

| n=219 | Clinical BSI  (BC-proven/probable BSI) | | Clinical BSI  (BC-proven/ probable/possible BSI) | |
| --- | --- | --- | --- | --- |
|  | Present | Not Present | Present | Not Present |
| mNGS Positive | 28 | 57 | 55 | 30 |
| mNGS Negative | 9 | 125 | 9 | 125 |
| Sensitivity/95%CI | 75.7% | 58.4% to 87.6% | 85.9% | 74.5% to 93.0% |
| Specificity/95%CI | 68.7% | 61.3% to 75.2% | 80.6% | 73.4% to 86.4% |
| Positive Predictive Value /95%CI | 32.9% | 23.4% to 44.1% | 64.7% | 53.5% to 74.6% |
| Negative Predictive Value /95%CI | 93.3% | 87.3% to 96.7% | 93.3% | 87.3% to 96.7% |
| Area Under the Curve /95%CI | 0.722 | 0.623 to 0.811 | 0.833 | 0.772 to 0.894 |

Abbreviations: BC, blood culture; BSI, bloodstream infection; mNGS, metagenomic next-generation sequencing

**Supplemental Figure Legends**

**Supplemental Fig. 1** The positivity distribution of mNGS and BC in diagnosing clinical BSI. a) Overall positive rate; b) Gram-negative bacteria; c) Anaerobic bacteria; d) Fungi; e) Gram-positive bacteria

Abbreviations: BC, blood culture; mNGS, metagenomic next-generation sequencing

**Supplemental Figures.**

**Supplemental Fig. 1**

| 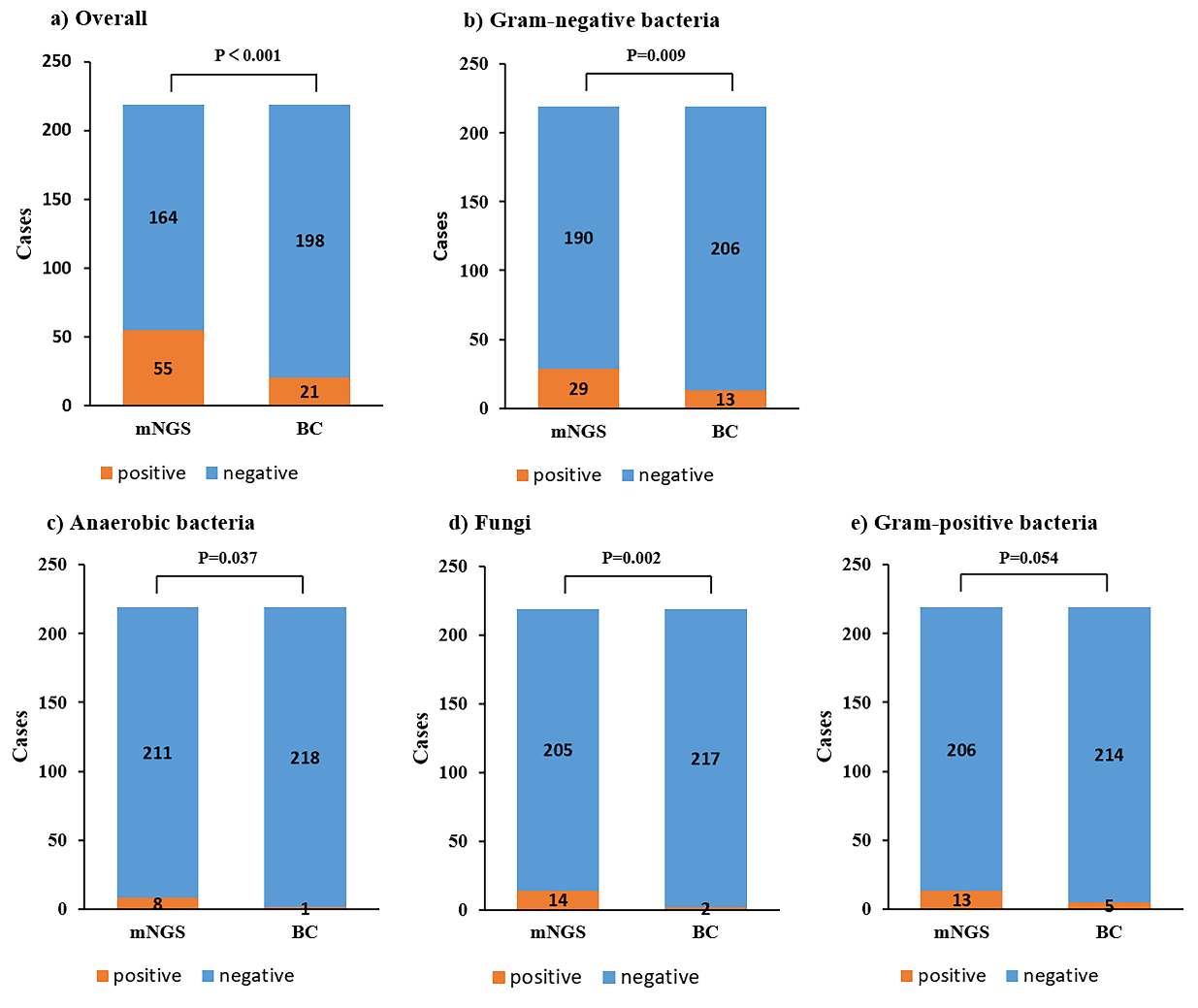 |
| --- |
